# Small incision lenticule extraction (SMILE) in the correction of myopic astigmatism: outcomes and limitations - an update

**DOI:** 10.1186/s40662-017-0091-9

**Published:** 2017-11-15

**Authors:** Jorge L. Alió del Barrio, Verónica Vargas, Olena Al-Shymali, Jorge L. Alió

**Affiliations:** 1 Cornea, Cataract and Refractive Surgery Unit, Vissum Corporación, Alicante, Spain; 2 Division of Ophthalmology, School of Medicine, Universidad Miguel Hernández, Alicante, Spain

**Keywords:** Small incision lenticule extraction, SMILE, Astigmatism, Refractive surgery

## Abstract

Small Incision Lenticule Extraction (SMILE) is a flap-free intrastromal technique for the correction of myopia and myopic astigmatism. To date, this technique lacks automated centration and cyclotorsion control, so several concerns have been raised regarding its capability to correct moderate or high levels of astigmatism. The objective of this paper is to review the reported SMILE outcomes for the correction of myopic astigmatism associated with a cylinder over 0.75 D, and its comparison with the outcomes reported with the excimer laser-based corneal refractive surgery techniques. A total of five studies clearly reporting SMILE astigmatic outcomes were identified. SMILE shows acceptable outcomes for the correction of myopic astigmatism, although a general agreement exists about the superiority of the excimer laser-based techniques for low to moderate levels of astigmatism. Manual correction of the static cyclotorsion should be adopted for any SMILE astigmatic correction over 0.75 D.

## Background

**Fig. 1:**
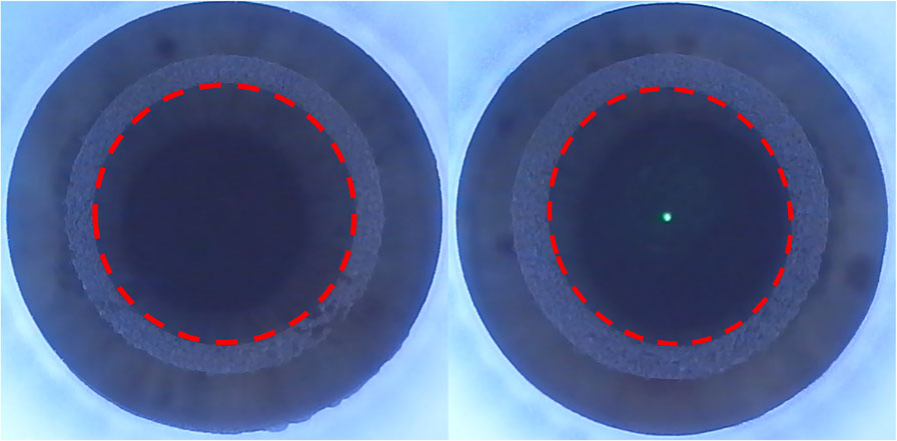
VisuMax dissection of the posterior refractive plane of the lenticule during a SMILE procedure of both eyes from the same patient. The left eye (left image) did not have any preoperative cylinder, while the right eye (right image) presented 1.75 D of the refractive cylinder. Note the oval shape of the dissection plane (red dotted line) in the astigmatic eye (right)

**Fig. 2:**
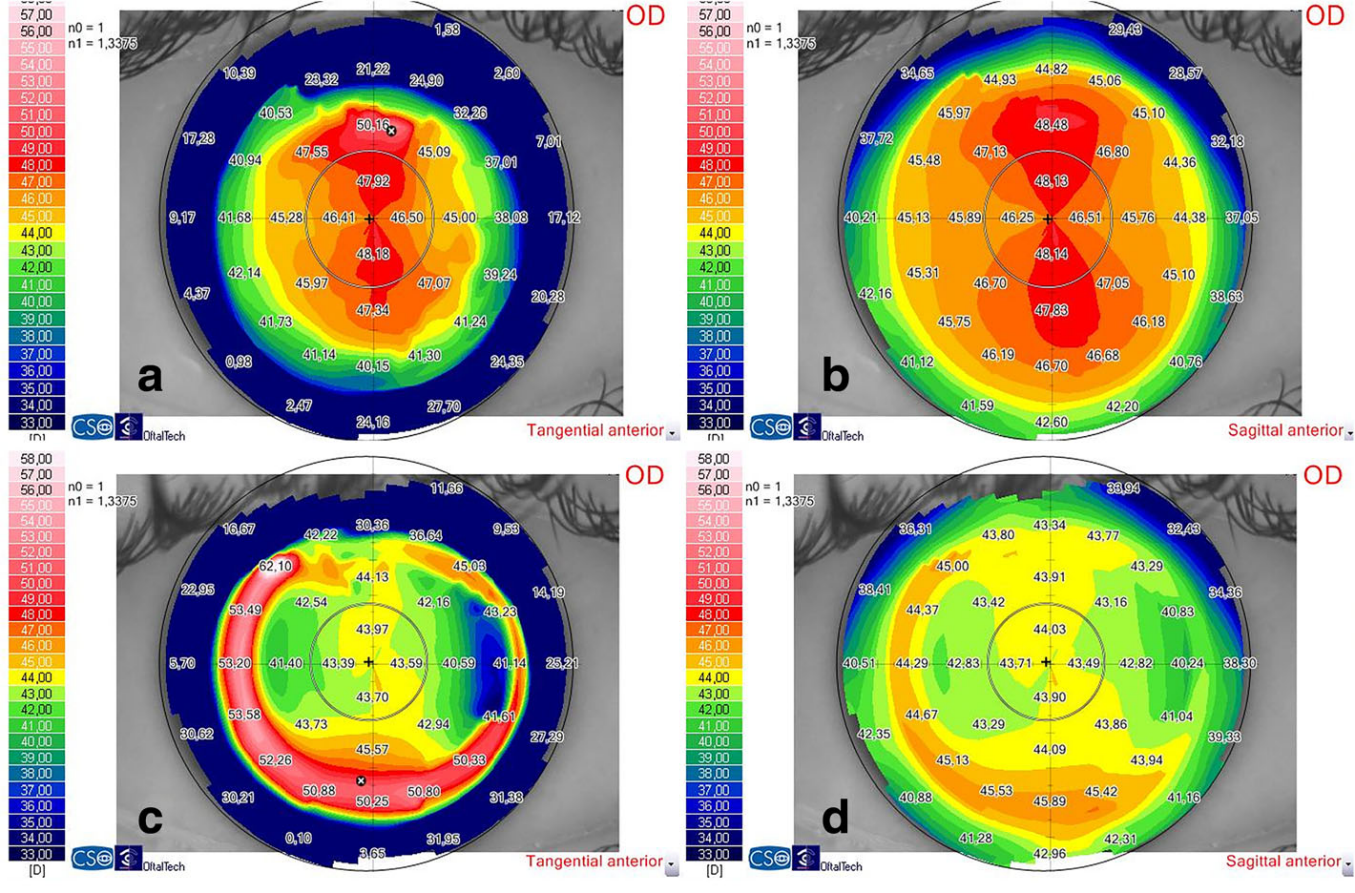
Topographic changes after a SMILE procedure in a 28-year-old patient with 2.75 D of the refractive cylinder. The figures **a** and **b** represent the tangential and sagittal anterior maps before surgery and the figures **c** and **d** are the same maps 6 months after the SMILE procedure. The patient had a residual refractive astigmatism of 1.00 D

Small Incision Lenticule Extraction (SMILE) was first introduced in 2011 as a flap-free intrastromal laser assisted refractive surgery technique for the correction of myopia and myopic astigmatism (Fig. 1), [1, 2] with a reported high efficacy, predictability, stability, and safety for myopic treatments together with a reduced risk of postoperative dry eye syndrome, enhanced postoperative corneal strength and lower amount of induced corneal aberrations [3–9]. However, the lack of cyclotorsion control on the VisuMax (Carl Zeiss Meditec, Germany) and the complete surgeon-dependent centration of the treatment have raised some concerns regarding the capability of SMILE to properly correct moderate or high levels of myopic astigmatism with the current commercially available technology (Fig. 2). Few papers report, by vector analysis, the outcomes for the treatment of myopic astigmatism, and very little evidence still exists regarding those cases associated with high cylinder. Moreover, we have to consider that even the excimer-based corneal refractive surgery techniques [surface ablation and laser in-situ keratomileusis (LASIK)] have demonstrated less effective and more difficult astigmatism correction compared with the treatment of simple spherical errors, and high astigmatic corrections seem to have a lower predictability and stability [10–14]. On the other hand, astigmatism ≤0.5 D doesn’t degrade visual acuity, suggesting that the visual benefit of the correction of this amount of astigmatism is limited [15].

Currently, SMILE is unsuitable for the treatment of simple or compound hyperopic astigmatism and current evidence does not support the treatment of mixed astigmatism with SMILE due to the lack of conclusive published data demonstrating its efficacy and safety for this type of refractive error [16].

The objective of this paper is to review the reported SMILE outcomes available in the peer-reviewed ophthalmic literature for the correction of myopic astigmatism associated with moderate or high cylinder (≥0.75 D) and its comparison with the outcomes reported with the excimer laser-based corneal refractive surgery techniques.

## Main text

### Material and methods

A PubMed review was performed, analysing all publications from 2010 to 2017 with the topic “SMILE correction of myopic astigmatism” (keywords: Small incision lenticule extraction, SMILE, astigmatism). Only published studies (in English – full text) specifically reporting astigmatic outcomes after SMILE correction of myopic astigmatism with a preoperative cylinder ≥0.75 D were included for this publication. Table 1 summarizes the outcomes reported in the different studies considered for this review. A meta-analysis of those outcomes was not the purpose of the present review. All studies that we reviewed were classified based on their scientific level of evidence according to the General Guidelines for Methodologies on Research and Evaluation of Traditional Medicine of the World Health Organization [17]. This classification is as follows: level Ia, evidence obtained from meta-analysis of randomized controlled trials; level Ib, evidence obtained from at least one randomized controlled trial; level IIa, evidence obtained from at least one well-designed controlled study without randomization; level IIb, evidence obtained from at least one well-designed quasi-experimental study; level III, evidence obtained from well-designed non-experimental descriptive studies, such as comparative studies, correlation studies and case-control studies; level IV, evidence obtained from expert committee reports or opinions and/or clinical experience of respected authorities.

**Table 1:** SMILE reported outcomes for the treatment of refractive myopic astigmatism with cylinder ≥0.75 D

Author	Procedure	N	Mean cylinder (D)	Efficacy index	Safety index	Predictability	Correction index	Difference vector	Magnitude of error	Angle of error ^b^	Follow up
Pedersen [20]	SMILE	101	−1.81	NA; 49% of eyes postop UDVA ≥ preop CDVA	NA; 9% lost one line in CDVA	70% of eyes within ±0.5 D of refractive cylinder	0.94	0.31	0.12	0.34	12 months
Chan [23]^a^	SMILE	54	−1.08	0.85; 76% achieved a UDVA of 20/25	0.97; 7.4% lost one line in CDVA	87% of eyes within ±0.5 D of refractive cylinder	0.858^a^	0.349^a^	−0.225^a^	6.38^a^	3 months
Chan [23]^a^	f-LASIK	57	−1.37	0.94; 95% achieved a UDVA of 20/25	0.98; 5.3% lost one line in CDVA	98.2% of eyes within ±0.5 D of refractive cylinder	1.020^a^	0.090^a^	0.037^a^	−0.72^a^	3 months
Zhang [24]	SMILE	42	−2.48	93% UDVA ≥20/25	0% lost CDVA lines	Mean postop refractive cylinder of −0.38 ± 0.42 D	0.88	0.36	−0.31	1.22	3 months
Zhang [24]	f-LASIK	22	−2.56	91% UDVA ≥20/25	0% lost CDVA lines	Mean postop refractive cylinder of −0.42 ± 0.45 D	0.94	0.42	−0.23	−1	3 months
Ganesh [27]	SMILE	81	−1.85	NA; 61% of eyes postop UDVA ≥ preop CDVA	NA; 0% lost one line in CDVA	86% of eyes within ±0.5 D of refractive cylinder	0.95	0.33	−0.108	4.09 (absolute AE)	3 months
Qian [30]^c^	SMILE	28 (25)	−1.35 (−2.50)	NA	NA	Mean postop refractive cylinder of −0.42 (−0.48) D	0.94 (0.94)	NA	NA	NA	6 months
Qian [30]^c^	LASEK	17 (14)	−1.27 (−2.55)	NA	NA	Mean postop refractive cylinder of −0.27 (−0.89) D	0.97 (0.77)	NA	NA	NA	6 months

*N* = number of eyes; *NA* = Information not available; *SMILE* = Small incision lenticule extraction; *LASEK* = laser-assisted subepithelial keratectomy; *f-LASIK* = femtosecond-assisted laser in-situ keratomileusis; *UDVA* = uncorrected distance visual acuity; *CDVA* = corrected distance visual acuity

^a^Outcomes reported for low and moderate astigmatism; the presented vector analysis is the specific one for the moderate astigmatism subgroup >1.5D of refractive cylinder; ^b^Arithmetic Angle of Error; ^c^Outcomes reported for moderate (1–2 D of cylinder) astigmatism in periods are shown the outcomes for the high (> 2 D) astigmatism subgroup

Astigmatism correction outcomes have been standardised for scientific publications for an easier comprehension and analysis by the reader as well as for an easier comparison with other reported results [18]. Also, astigmatism might be analysed as a vector composed of a magnitude and an axis. The vectorial analysis of Alpins allows us to assess the effectiveness of the astigmatic treatment by determining the amount of correction and whether this correction was on axis or off axis [19]. This method determines 3 fundamental vectors: target-induced astigmatism vector (TIA), which is the astigmatic change the surgery was intended to induce; surgically induced astigmatism vector (SIA), which is the real astigmatic change achieved by the surgery; and the difference vector (DV), which is the additional astigmatic change required to meet the intended target of the initial surgery. In a perfect astigmatic correction, the TIA and SIA have the same magnitude and the difference vector must be zero. Using these three values, the Alpins method obtains other indices, such as the magnitude of error (ME), which is the arithmetic difference between the magnitudes of the SIA and the TIA (positive for overcorrections and negative for undercorrections), and the angle of error (AE), which is the angle described by the vectors of the SIA and TIA (positive or negative if the achieved correction is counterclockwise or clockwise to the intended axis, respectively). The Alpins method also calculates the correction index (CI), which is the ratio obtained by dividing the SIA by the TIA. The preferable CI value is 1.0; values larger than 1.0 indicate that overcorrection occurred while values lower than 1.0 indicate the occurrence of undercorrection. In addition, the Alpins method determines the index of success (IOS), that reflects the proportion of residual astigmatism and it is calculated by dividing the difference vector by the TIA; the preferable value is zero.

## Results

We identified a total of five studies where SMILE astigmatic outcomes with a preoperative cylinder of at least 0.75 D were independently reported (Table 1). All evaluated papers had a level IIb of scientific evidence according to the General Guidelines for Methodologies on Research and Evaluation of Traditional Medicine of the World Health Organization [17].

Pedersen et al. recently published their one-year postoperative SMILE outcomes for the treatment of myopic astigmatism with up to 4.00 diopters of cylinder (101 eyes), in what is probably the most important paper on this specific topic to date (Table 1) [20]. In their series, 70% and 94% had a postoperative refractive astigmatism of less than 0.50 D and 1.00 D respectively and only 49% of eyes achieved an unaided distance visual acuity (UDVA) equal or better than the preoperative corrected distance visual acuity (CDVA) (83% within one line of the preop CDVA). Nine percent of eyes lost one Snellen line in CDVA while 55% experience no change and 35% improved by one or more Snellen lines. No eye lost 2 or more lines of CDVA. Vector analysis showed a with-the-rule undercorrection with a mean difference vector of 0.31 D. Approximately, an undercorrection of 11% per diopter of attempted correction was observed. They also described a minor counterclockwise rotation of the axis (mean angle of error of 0.34°). Overall, the CI at 12 months postop was 0.94, with no significant changes during the postoperative period. Something important noted in this paper is the fact that the magnitude, angle of error and the amount of undercorrection were progressively higher with increasing preoperative astigmatism. Finally, they reported that RMS of coma and HOAs increased significantly after surgery, but remained unchanged in the postoperative period [20]. In a previous paper from the same group evaluating the postoperative astigmatism correction three months after SMILE, they also reported this undercorrection tendency, being 13% per diopter of attempted correction after treatment of low astigmatism (mean −1.22 D of cylinder) and 16% after treatment of high astigmatism (mean −3.22 D of cylinder) [21]. Zhang et al. reported comparable results, observing a 12-month postoperative undercorrection with minor counterclockwise axis error in eyes treated for low to moderate astigmatism [22]. Moreover, they demonstrated a significant negative correlation between the CI and TIA values. In this study, preoperative cylinders as low as −0.25 D were included, so their reported outcomes have not been further considered in the present review.

Comparative studies between femto-LASIK and SMILE for the treatment of myopic astigmatism have been also performed. Chan et al. reported that UDVA at 1 and 3 months were significantly better in the LASIK group compared with the SMILE group (Table 1).[23] Additionally, the postoperative cylinder was higher in the SMILE group (*p* < 0.001). Vector analysis did not show significant differences in target-induced astigmatism but the surgically-induced astigmatism was significantly lower in the SMILE group while the difference vector and absolute angle of error were significantly higher in the SMILE group (Table 1) [23]. These differences were greater in those eyes with a moderate preoperative cylinder (mean TIA 1.58 D) compared with those presenting a low preoperative cylinder (mean TIA 0.54 D). Vector analysis for the SMILE eyes with preoperative moderate cylinder 3 months after surgery was: DV of 0.34 D; ME of −0.22 D (undercorrection); AE of 6.38°; CI of 0.86 (Table 1).

Zhang et al. compared the refractive outcomes after wavefront-guided LASIK and SMILE for moderate and high myopic astigmatism as well (Table 1) [24]. In the moderate astigmatism group, CI was 1.04 after wavefront-guided LASIK and 0.88 after SMILE (*p* < 0.05). However, in the high-astigmatism group, no significant differences in the correction index between both techniques were reported [24]. In a similar fashion as Chan et al., they observed that the average AE value was negative after LASIK and positive after SMILE, where these differences were statistically significant. They postulated that it could be justified by the unconscious eyeball movement shortly before activating vacuum suction during SMILE. In concordance with the already published literature, a clear tendency towards undercorrection was observed in the high-astigmatism group after both surgeries.

Khalifa et al. published a prospective consecutive comparative randomized clinical trial comparing the efficacy of the astigmatic correction achieved with wavefront-guided LASIK and SMILE in eyes with myopia and different levels of myopic astigmatism [25]. They observed statistically significant differences in all analysed outcome parameters between both techniques in favour of femto-LASIK, and in concordance with other authors, a significantly more positive AE in the SMILE group and a positive significant correlation between the magnitude of the preoperative cylinder and the postoperative difference vector. However, the authors did not independently analyse those eyes with moderate or high astigmatism from those with low astigmatism. In fact, they evaluated eyes without any refractive astigmatism (0.0 D) with eyes having up to 4 D of cylinder (resulting in a mean refractive cylinder <1.5 D in both groups) without analysing subgroups. Considering this potential bias, their outcomes have not been further considered in the present review study. Similar conclusions, but with the same limitation, have been recently published by Kanellopoulos et al. on a prospective, randomized study comparing the safety and efficacy of topography-guided femto-LASIK versus SMILE [26].

In an attempt to improve SMILE outcomes for the treatment of myopic astigmatism, Ganesh et al. recently studied a practice commonly conducted by SMILE surgeons, the manual compensation of the intraoperative torsional error guided by the preoperative limbal marking [27]. Briefly, preoperative limbal marks at 0° and 180° using a dye in the upright position are used to detect the extent of cyclotorsion (if any) under the Visumax using as a guide the reticule of the right eyepiece after suction has been applied. Then, existing cyclotorsion is manually compensated by gentle rotation of the contact glass to align the horizontal marks on the eye to the 0° to 180° axis of the VisuMax right eyepiece reticule. In their series, 81% of eyes showed a cyclotorsion ≤5° (18% of eyes did not show any), 17.6% between 6° and 10°, and 1.2% of eyes ≥10°. Reported outcomes are shown in Table 1. As expected, a higher tendency towards undercorrection was observed in the high astigmatism subgroup (> 1.5 D). However, in the vector analysis comparing the moderate and high astigmatism subgroups, only the ME resulted being significantly higher in the latter, while DV and CI did not show significant differences. Absolute AE and IOS were significantly higher in the low cylinder subgroup compared to the high cylinder one [27].

With recent excimer laser advances like flying-spot lasers, larger optical zones and wavefront-guided treatments, photorefractive keratectomy (PRK) have shown to offer similar outcomes to LASIK for myopic astigmatic correction [11, 28, 29]. However, to the best of our knowledge, only one retrospective study compares the efficacy of SMILE and a surface ablation treatment [specifically laser-assisted subepithelial keratectomy (LASEK)] in correcting myopic astigmatism (Table 1) [30]. They observed that for low astigmatism (< 1 D) LASEK performed significantly better than SMILE (with significantly lower remaining refractive cylinder), reporting a CI closer to 1.0 for LASEK. For moderate astigmatism (1–2 D), LASEK showed slightly better results than SMILE, but no significant differences were found between both techniques. On the other hand, for high astigmatism (> 2 D), the remaining refractive astigmatism was much higher for LASEK than for SMILE, and the CI was much lower for LASEK.

## Discussion

Considering all published literature, we can conclude that SMILE presents an acceptable predictability, efficacy, stability and safety for the correction of moderate to high myopic astigmatism [20–27]. However, a clear tendency towards undercorrection has been reported by several authors, where this undercorrection is more pronounced when the preoperative astigmatism is greater [20–26]. Accordingly, Pedersen and Ivarsen et al. suggested that current treatment nomograms may require adjustment by 10% in the magnitude of astigmatism correction [20, 21].

Compared with femto-LASIK, SMILE offers a less favourable astigmatic correction.[23–26] Cyclotorsion of the eye from standing to supine position is a common concern when correcting astigmatism, as a cyclotorsional error of only a few degrees is a source of astigmatic undercorrection [31, 32]. The lack of an automated cyclotorsion control and centration in the Visumax, as we already have in the latest excimer laser platforms, could justify some of these differences, since centration and alignment of the treatment is subjective and more variable in SMILE, as it purely depends on the patient’s fixation and surgeon’s application of the suction contact glass on the eye. On the other hand, SMILE centration has not been proven to be worse than in excimer based treatments as long as a depurate technique is applied and careful attention to treatment centration is paid after applying the suction and before starting the treatment [33]. It has been reported that during excimer refractive surgery, as many as 38% of eyes rotate more than 5° from the seated position to the supine position (static rotation) and as many as 68% of eyes rotate more than 2° from the seated position until the end of the laser treatment (static and dynamic rotation) [34]. Although dynamic cyclotorsion should not be an issue for SMILE due to the eye fixation during laser treatment, static cyclotorsion should be compensated as correct axis alignment and pupil centration are critical elements. Causes of cyclotorsion misalignment include rotation of the head and body under the laser, ocular torsion caused by the vestibular system and unmasking of cyclophoria. Monocular fixation has been shown to allow significant cyclotorsion as well. When ocular cyclotorsion of more than 2° occurs and is not corrected, astigmatism correction can be influenced and significant aberrations can be induced [34]. Theoretically, a 4-degree difference would result in a 14% undercorrection of astigmatism, a 6-degree difference would result in a 20% undercorrection of astigmatism, and a difference of 10° would result in a 35% undercorrection [35]. Bharti and Bains reported a statistically significant lower cylinder dispersion and mean manifest refractive cylinder after ablations using active cyclotorsion error correction in the treatment of myopia and myopic astigmatism.[36] Ganesh et al. reported that in their SMILE series, nearly 20% of eyes showed a cyclotorsion of more than 5° [27]. They demonstrated better SMILE astigmatic outcomes with the manual compensation of the static cyclotorsion guided by preoperative limbal marks. This technique (not officially approved by Zeiss yet), in the author’s personal experience, is vulnerable to errors in several areas: while placing the limbal marks at the limbus and at the paracentral cornea, while adjusting the patient’s head under the VisuMax to match the horizontal eyepiece reticule and while rotating the contact glass for the final axis adjustment. Moreover, ink corneal marks are not always easy to see once suction has been applied if the patient has a dark iris colour. Also, gentle and small rotations of the contact glass should be attempted to avoid an undesirable loss of suction. However, as previously discussed, static cyclotorsion compensation is a key element in the correction of astigmatism, and this technique although not perfect, should be adopted for any astigmatic correction over 0.75D until a proper iris/limbal registration for an automated cyclotorsion control and pupil centration is available for VisuMax.

Conversely, if we compare reported SMILE astigmatic outcomes with those reported with femto-LASIK using the latest excimer laser technology for high myopic astigmatism (> 3 D), we will observe a generalized undercorrection tendency with both techniques and no significant differences regarding the efficacy, safety and predictability. In fact, our group published a study in 2013 that evaluated the safety and efficacy of femto-LASIK using an excimer laser platform (Amaris, Schwind) with fast repetition rate, optimized aspheric ablation profile, and cyclotorsion control to treat myopic astigmatism with high cylinder (mean preop cylinder of −3.64 D) [12]. In that study, 87% and 97% of eyes had a postoperative SE within ±0.50 D and ±1.00 D of the intended correction, respectively, with a 7.5% retreatment rate. Furthermore, the Alpins vectorial analysis DV was 0.52, ME was −0.28, arithmetic AE was −0.49° and CI was 0.91. Ivarsen et al. reported very similar results using the AMEL-80 flying-spot laser (Carl Zeiss Meditec AG) with a mean preoperative cylinder of −3.9 D, and observed an undercorrection of about 21% per diopter [37]. These studies have a mean preoperative cylinder higher than the one reported by SMILE papers, but considering Ivarsen et al. and Zhang et al. studies [21, 24] with the highest preoperative cylinder using SMILE (−3.22 and −2.48 D respectively), we can observe that their reported outcomes are equivalent to those observed by our group and Ivarsen et al. for high cylinder cases. Considering all the above-mentioned studies, femto-LASIK seems superior to SMILE for low and moderate levels of astigmatism but for the treatment of high astigmatism, both techniques suffer an important drop in their efficacy and predictability and seem to have a more equivalent outcome. However, while retreatments are straightforward for LASIK (early flap lift is safe, effective and with an immediate visual recovery) the same cannot be said for SMILE. The sub-cap-lenticule-extraction for refractive enhancement after a primary SMILE (which uses the previous interface as the new superior plane for a new lenticule) [38] is not standardized and its efficacy and safety are not properly established thus, SMILE conversion into LASIK by the opening of the cap with the circle mode of the VisuMax is probably the preferable method for retreatments. However, this option is not always possible if the expected residual stromal bed is unacceptable (common situation if a significant ametropia was previously treated). For those cases, PRK over the cap is the current treatment choice. SMILE enhancements with PRK + MMC have been reported to have acceptable outcomes, [39] although the patient should be advised regarding the long postoperative visual recovery. In this study, preoperative astigmatism over 3.00 D was identified as a significant risk factor for enhancement after SMILE (odds ratio 3.06) [39].

There is a general agreement among published literature that while LASIK shows consistently negative values for the angle of error by vectorial analysis, SMILE shows positive values in all studies (Table 1). That is translated into a repeated counterclockwise deviation of the intended axis. Mild torsional eye movements at the time of applying the suction may justify this finding.

Finally, an accurate refraction plays a critical role in the refractive surgical outcome as it can be a cause of undercorrection and this could partially justify the previously reported outcomes of SMILE in astigmatism correction. Reinstein et al. reported a high interobserver (optometrists and surgeons) reproducibility of manifest refraction independently of the magnitude of the refractive error by using a standardized refraction protocol, wherein the sphere and cylinder is refined once the patient can read the 20/20 line by adding in steps of +0.25 diopters of sphere and −0.25 diopters of cylinder, with constant refinement of the axis with the Jackson Cross [40]. With this standardized protocol, the mean dioptric power difference was 0.21 D between surgeons and optometrists.

## Conclusion

In conclusion, SMILE shows acceptable outcomes for the correction of myopic astigmatism, although a general agreement exists about the current superiority of the excimer laser-based techniques for the treatment of low and moderate levels of astigmatism. For cylinders over 3 D, both techniques suffer an important loss of efficacy and predictability but enhancements after LASIK are easier and provide a faster visual recovery. Important improvements in SMILE astigmatic outcomes are expected in the next few years once an automated centration and cyclotorsion control are available for VisuMax.

Therefore, given the reviewed literature, the following recommendations may be followed to improve our SMILE astigmatic outcomes: 1) manual correction of the static cyclotorsion for any astigmatic correction over 0.75 D [27]; 2) 10% correction increment over the original refractive cylinder value [20, 21]; 3) use of a standardized refraction protocol to refine the cylinder measurement since incorrect preoperative refraction can lead to postoperative residual refractive errors [40].
